# The complete chloroplast genome of *Narcissus* ‘Pink Charm’

**DOI:** 10.1080/23802359.2021.1984329

**Published:** 2021-09-27

**Authors:** Ana Bian, Luanmei Lu, Dongming Pan

**Affiliations:** a Department of Biological Science and Biotechnology, Minnan Normal University, Key Laboratory of Landscape Plants with Fujian and Taiwan Characteristics of Fujian Colleges and Universities, Zhangzhou, Fujian, China; b College of Horticulture, Fujian Agriculture and Forestry University, Fuzhou, Fujian, China

**Keywords:** *Narcissus* ‘Pink Charm’, complete chloroplast genome, phylogenetic analysis

## Abstract

The first complete chloroplast (cp) genome of *Narcissus* ‘Pink Charm’ was sequenced and characterized using Illumina paired-end data. The assembled cp genome was 159,988 in length with a GC content of 37.82%. A total of 137 genes were annotated, consisting of 91 protein-coding genes, eight ribosomal RNA genes, and 38 transfer RNA genes. The phylogenetic position based on the cp genome data revealed that *Narcissus* ‘Pink Charm’ is more closely related to *Narcissus poeticus* than other relative species.

Subject classification codes: include these here if the journal requires them *Narcissus* ‘Pink Charm’ is bulbous herbaceous perennials belonging to the Amaryllidaceae family, with linear leaves and solitary flowers, with six spreading perianth segments and a cup-shaped corona. *Narcissus* ‘Pink Charm’ is a large-cupped cultivar, *Narcissus* ‘Pink Charm’ is popular as a cut flower, garden flower, and pot plant, owing mainly to its unique flower with creamy-white flowers and with a cream trumpet that is prominently edged with salmon-pink. Previous studies have been focused on biologically active compounds, volatile compounds, and in the bulb of *Narcissus* ‘Pink Charm’ (Li et al. 2018; Akram et al. 2021). Up to now, there are no published *Narcissus* ‘Pink Charm’ chloroplast genomes. Here, the complete chloroplast genome sequence of *Narcissus* ‘Pink Charm’ was reported to provide an effective use of genetic resources in breeding programs.

The plant sample was collected from Gongyashan Forest Park, Huaan Town, Zhangzhou (Fujian, China, 117°53′50″E, 25°02′30″N). Total genomic DNA was isolated from fresh leaves following the CTAB DNA extraction protocol (Li et al. 2013). Paired-end Illumina genomic library was prepared. The voucher specimens of *Narcissus* ‘Pink Charm’ were deposited at the laboratory of the Department of Biological Science and Biotechnology, Minnan Normal University, Zhangzhou (accession number: No.MNU001; URL: https://bio.mnnu.edu.cn/info/1110/2223.htm) (Ana Bian, Email: 549511030@qq.com). Whole-genome was sequenced on an Illumina Hiseq 2000 platform (Illumina, San Diego, CA, USA) at Beijing Genomics Institute (BGI, Shenzhen, China) and yielded 3.5 G of raw data. Quality control was performed to use the FastQC program (Andrews 2014), and then clean reads of around 3.0 G were assembled to chloroplast genomes using SPAdes version 3.11.0 (Bankevich et al. 2012). Annotation was completed by the online program GeSeq (Tillich et al. 2017) with the reference chloroplast genome of *Narcissus tazetta*. Chinensis (GenBank: MN432153) as reference. The chloroplast genome of *Narcissus* ‘Pink Charm’ was deposited in Genbank with accession number MW672399.

The circle genome of is 159,988 bp in size with an overall GC content of 37.8%. It comprises a large single-copy region (LSC) of 86,430 bp, a small single-copy region (SSC) of 16,562 bp, and a pair of inverted repeat regions (IR) of 28,498 bp. In addition, 137 genes were identified, including 91 protein-coding genes, 38 transfer RNA genes (tRNA), and eight ribosomal RNA genes (rRNA). Among all unique genes, 15 genes tRNA(*trn*K-UUU, *rps*16, *trn*G-UCC, *atp*F, *rpo*C1, *trn*L-UAA, *trn*V-UAC, *pet*B, *pet*D, *rpl*16, *rpl*2, *ndh*B, *trn*I-GAU, *trn*A-UGC, and *ndh*A) contained one intron. Two genes (*clp*P and *ycf*3) contained two introns. The chloroplast genomes of 27 species plants were used to construct phylogenetic trees using maximum-likelihood (ML) methods and performed to use the RaxML software v 8.2.9 with 1000 bootstrap replicates on the CIPRES Science Gateway v. 3.3 (Stamatakis 2014). The ML tree showed that *Narcissus* ‘Pink Charm’ was grouped to *Narcissus poeticus* in the *Narcissus* genus Amaryllidaceae family (Figure 1).

**Figure 1.**
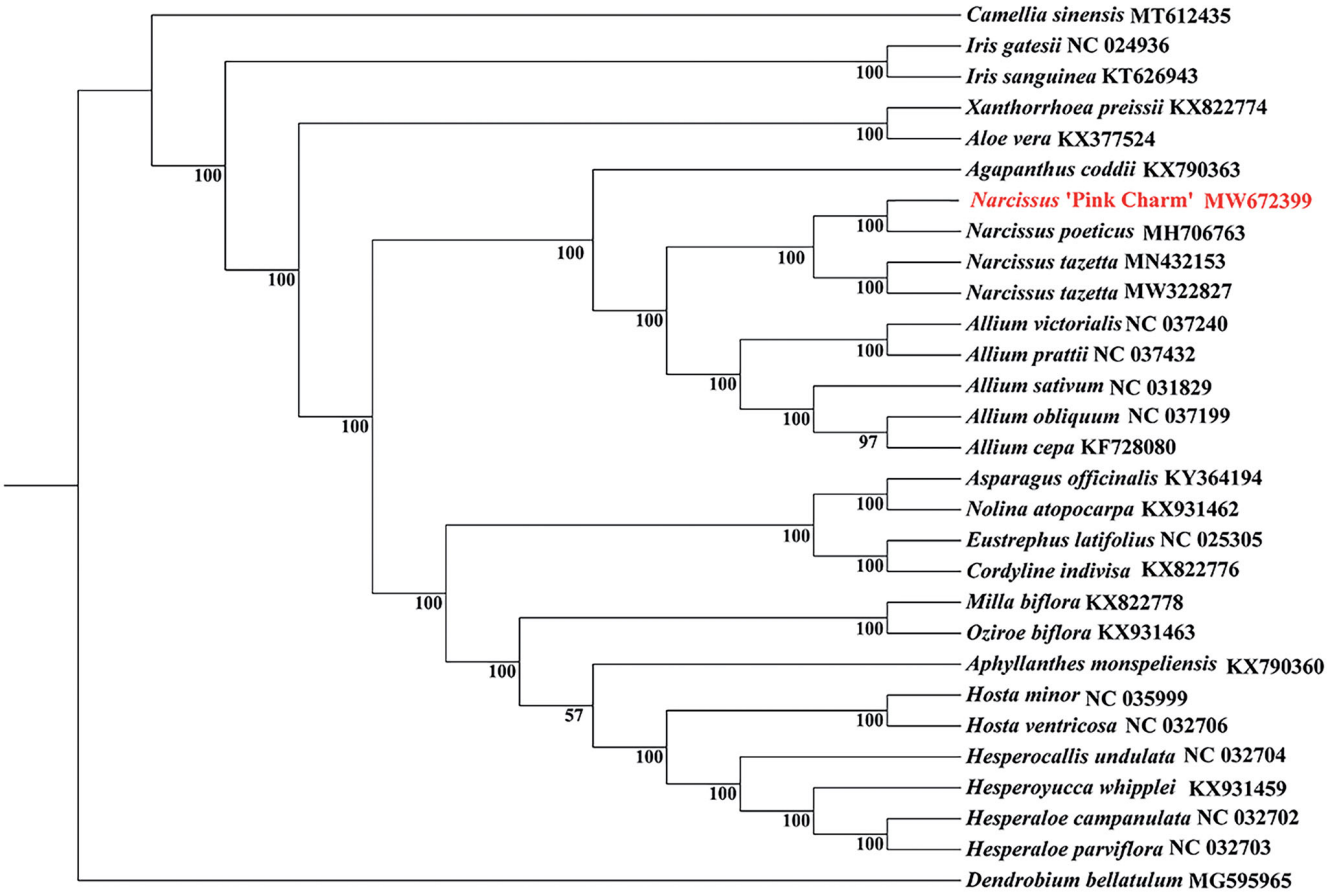
The maximum likelihood (ML) phylogenetic tree was constructed based on complete plants chloroplast genomes data of the 28 species.

## Data Availability

The data that support the findings of this study are openly available in GenBank of NCBI at https://www.ncbi.nlm.nih.gov, reference number MW672399. The raw data of sequence were submitted to NCBI, and BioProject, SRA, and Bio-Sample number are PRJNA719679, SAMN18614431, and SRR14141451, respectively.
